# *Almeidea* A. St.-Hil. Belongs to *Conchocarpus* J.C. Mikan (Galipeinae, Rutaceae): Evidence from Morphological and Molecular Data, with a First Analysis of Subtribe Galipeinae

**DOI:** 10.1371/journal.pone.0125650

**Published:** 2015-05-07

**Authors:** Carla Poleselli Bruniera, Jacquelyn A. Kallunki, Milton Groppo

**Affiliations:** 1 Departamento de Botânica, Instituto de Biociências, Universidade de São Paulo, São Paulo, São Paulo, Brazil; 2 The New York Botanical Garden, Bronx, New York, United States of America; 3 Departamento de Biologia, Faculdade de Filosofia, Ciências e Letras de Ribeirão Preto, Universidade de São Paulo, Ribeirão Preto, São Paulo, Brazil; The National Orchid Conservation Center of China; The Orchid Conservation & Research Center of Shenzhen, CHINA

## Abstract

Subtribe Galipeinae (tribe Galipeeae, subfamily Rutoideae) is the most diverse group of Neotropical Rutaceae, with 28 genera and approximately 130 species. One of its genera is *Almeidea*, whose species are morphologically similar to those of the genus *Conchocarpus*. Species of *Almeidea* occur in the Atlantic Rain Forest of Eastern Brazil, with one species (*Almeidea rubra*) also present in Bolivia. The objective of this study was to perform a phylogenetic analysis of *Almeidea*, using a broader sampling of Galipeinae and other Neotropical Rutaceae, the first such study focused on this subtribe. To achieve this objective, morphological data and molecular data from the nuclear markers ITS-1 and ITS-2 and the plastid markers *trnL-trnF* and *rps16* were obtained. Representatives of eight genera of Galipeinae and three genera of Pilocarpinae (included also in Galipeeae) and *Hortia* (closely related to Galipeeae) were used. Five species of *Almeidea* and seven of *Conchocarpus* were included, given the morphological proximity between these two genera. Individual (for each molecular marker) and combined phylogenetic analyses were made, using parsimony and Bayesian inference as optimization criteria. Results showed Galipeinae as monophyletic, with the species of *Almeidea* also monophyletic (supported by the presence of pantocolporate pollen) and nested in a clade with a group of species of *Conchocarpus*, a non-monophyletic group. Additionally, *C*. *concinnus* appeared in a group with *Andreadoxa*, *Erythrochiton*, and *Neoraputia*, other members of Galipeinae. As a result, *Conchocarpus* would be monophyletic only with the exclusion of a group of species related to *C*. *concinnus* and with the inclusion of all species of *Almeidea* with the group of species of *Conchocarpus* that includes its type species, *C*. *macrophyllus*. Thus, species of *Almeidea* are transferred to *Conchocarpus*, and the new combinations are made here.

## Introduction

Rutaceae is a large, predominantly tropical and subtropical family, consisting of 150–164 genera and 1500–2000 species, with three main centers of diversity: Tropical America, southern Africa, and Australia [1–5]. The family has long been economically important for edible fruits (especially *Citrus*, the oranges, lemons, tangerines, etc.), aromatic oils (*Boronia* and *Ruta*), drugs (e.g., *Pilocarpus*, source of pilocarpine, used in the treatment of glaucoma), and bitter beverages used to treat fevers (*Angostura*, *Galipea*). Many species are used as timbers (*Flindersia*, *Zanthoxylum*, *Balfourodendron*, *Euxylophora*), and more recently, the antimicrobial and antifungal properties of rutaceous compounds have being exploited and proven to be medicinally useful (e.g., [6–8]). Given their great diversity in morphological characteristics that include a variety of habits, flowers, and fruits, allied with a broad geographic distribution, Rutaceae have been traditionally divided into subfamilies, tribes, and subtribes, following the classifications of Engler ([9–11], see [12, 13] for a detailed discussion of the these groups). New subfamilial realignments have been recently published, and number of subfamilies varies from two to four [5, 14, 15]. Within the subfamily Rutoideae, the largest of the subfamilies sensu [14], the tribe Galipeeae comprise two subtribes, the Pilocarpinae and the Galipeinae, the latter being by far the most diverse group of Neotropical Rutaceae, with 28 genera and approximately 130 species [13]. Most of the Galipeinae occur in the Brazilian Atlantic Rain Forest, with some groups reaching the Guianas, the Andes, and Central America [13], mostly in the understory of moist forests [3].

**Fig 1:**
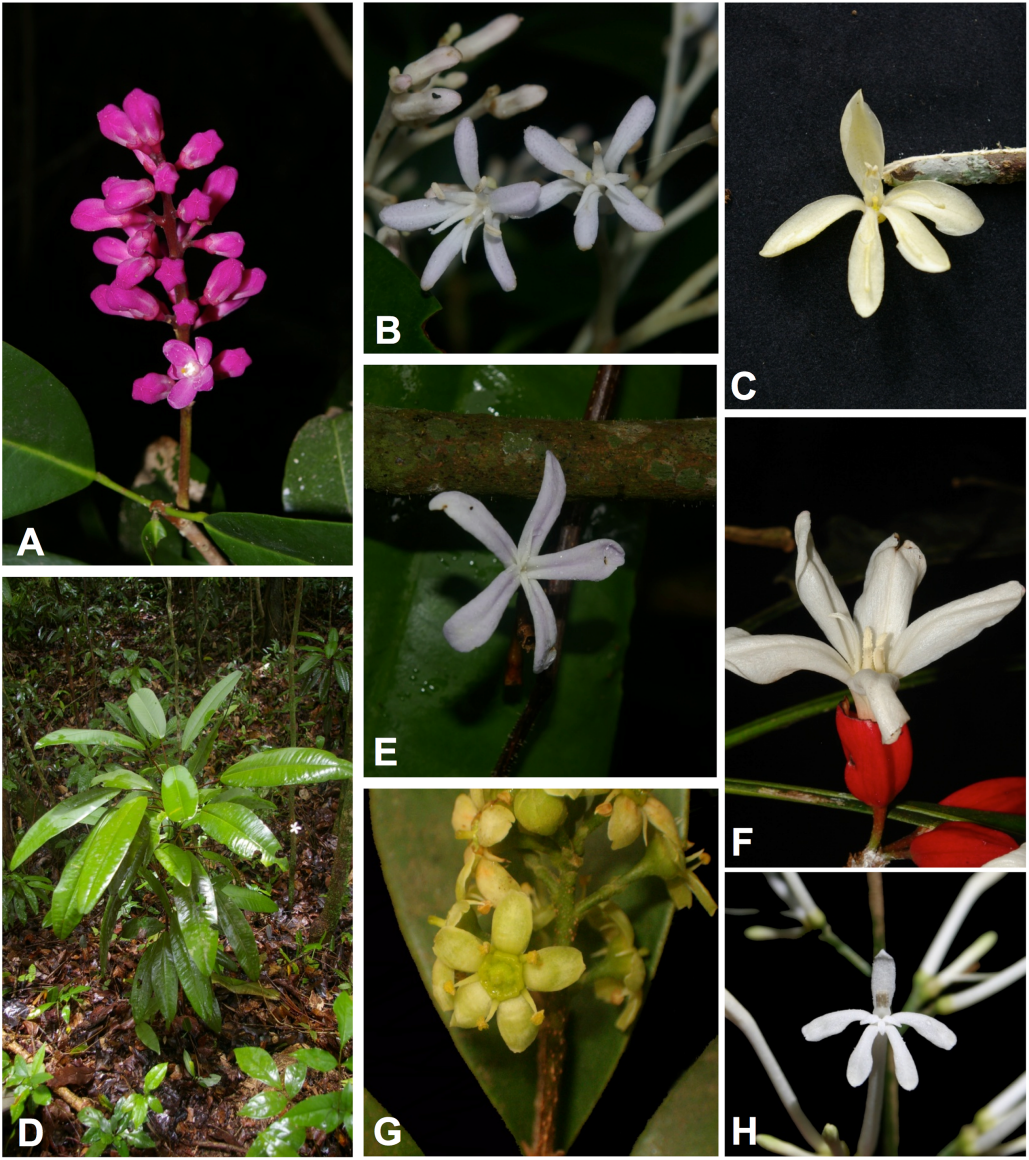
Photos of some species used in this study. A. Inflorescence of *Almeidea rubra*. B. Flowers of *Almeidea albiflora*. C. Flower of *Andreadoxa flava*. D. Habit of *Conchocarpus macrophyllus*. E. Flower of *Conchocarpus macrophyllus*. F. Flower of *Erythrochiton brasiliensis*. G. Flower of *Esenbeckia grandiflora* H. Flower of *Galipea jasminiflora*. All by Milton Groppo except F (by Cláudio N. Fraga) and H (by Flávio A. Bonatti).

Galipeinae encompass Neotropical Rutaceae with flowers mostly zygomorphic, a more or less tubular corolla, union of the filaments to a corolla tube, reduction of fertile stamens from five to two, basally appendaged anthers, and plicate cotyledons [10, 16] (see Fig 1 for some representatives); however, there are exceptions to all the morphological characteristics cited above. Additionally, base chromosome number [17] and pollen morphology [18] are very diverse in this group.

Galipeinae are taxonomically heterogeneous, including 10 monospecific genera (*e*.*g*., *Adiscanthus*, *Andreadoxa*, *Desmotes*, *Euxylophora*, *Lubaria*), as well as polymorphic genera (e.g. *Conchocarpus* with 48 species [16, 19]), with most of them occurring in the Brazilian Atlantic Rain Forest, but also in Amazonia and Central America, in other biomes. The circumscription and internal relationships of Galipeinae are currently under study by the authors.

One of the recognized genera of Galipeinae is *Almeidea* (Fig 1A and 1B), characterized as shrubs or treelets from forest understorey, with 1-foliolate, alternate leaves, a (sub)terminal thyrse and pink, lilac or white (in *A*. *albiflora*, recently described [20]) flowers with five stamens, of which up to three can be modified into staminodes (lacking anthers). Five species, all endemics to Brazilian Atlantic Rain Forest, were recognized in the last revision of the genus [21], but since then, collections of *A*. *rubra* from Bolivia have been identified.

*Almeidea* are morphologically similar to *Conchocarpus* (Fig 1D and 1E) but differ from the latter by their free petals (vs. coherent or connate in *Conchocarpus*) and, with few exceptions, by their pink or lilac color (vs. creamy-white). *Conchocarpus* are delimited by a combination of character states, with some of these states also present in other genera of Galipeinae [16], including in *Almeidea*. The similarity between *Almeidea* and *Conchocarpus* is reflected in the frequent misidentification of herbarium specimens in a vegetative state or fruiting condition. In a previous phylogenetic analysis of the subtribe using the plastid markers *trnL-trnF* and *rps16* [22], the single species of *Almeidea* and the three species of *Conchocarpus* that were included appeared in the same clade together with a few other genera from Galipeinae.

The objective of the present study is, therefore, to perform the first phylogenetic analysis to be focused on the Galipeinae and to include a greater number of species of these two genera as well as of related genera that have not yet been included in such a study, primarily to test the monophyly of *Conchocarpus* and its relationship with *Almeidea*. The results obtained will provide a framework for taxonomic decisions about the genera cited above, a base to support discussion of putative morphological synapomorphies of the groups, and a first step to a better understanding of the phylogeny of the Galipeinae and the Galipeeae.

## Material and Methods

### Taxa studied

All taxa included in this study belong to subfamily Rutoideae (as circumscribed in [14]). The Galipeinae were represented by all five currently recognized species of *Almeidea* [20, 21, 23], seven of *Conchocarpus*, and one each of *Erythrochiton*, *Galipea*, *Neoraputia*, *Rauia*, and *Ravenia* (the list of all species names and authors are listed in S1 and S2 Text). Given the wide geographic range of *Almeidea rubra*, individuals from three populations (two from Espírito Santo and Minas Gerais states, both in Brazil, and one from Bolivia) were included. The Pilocarpinae were represented by two species of *Esenbeckia* and one each of *Metrodorea* and *Pilocarpus*. In addition, one species of *Hortia* (a genus assigned traditionally to the tribe Toddaliinae of the subfamily Toddalioideae, nowadays included in Rutoideae) was also included because it was clustered with *Adiscanthus* (not sampled here), a genus of the Galipeinae, in a previous study [14]. One species of *Zanthoxylum* (tribe Zanthoxyleae) was used as outgroup in all analyses. The newly generated sequences were deposited in GenBank and the respective accession numbers are cited in S1 Text. Voucher specimens from which molecular sequences and morphological data were obtained were deposited at SPF and SPFR herbaria (herbarium acronyms according to [24]) and are summarized in S1 and S2 Text, respectively.

### Morphological analysis

The morphological analysis was based on macromorphological and pollen characters. Characters were codified according to procedures described in [25] and [26], and definition of morphological terms follows those in [16] and [27]. A list of all 35 characters and their respective states are in S3 Text, and the corresponding matrix is shown in S1 Table.

Data for morphological analysis were taken from specimens deposited at ALCB, BHCB, CEPEC, ESA, GFJP, HPL, HRB, HUEFS, LPB, MBM, MBML, MO, NY, RB, SP, SPF, SPFR and UPCB—herbarium acronyms are according to [24]. Macromorphological character states of leaves, flowers, and fruits were determined by direct observation of dry, fixed, or fresh samples or from descriptions of the taxa in literature (e.g., [16, 19–21, 28–36]). The matrix of morphological data was built with the software NDE 0.5.0 [37]. Voucher specimens from which morphological data were obtained are summarized in S2 Text.

The pollen characteristics of many taxa included in the analysis had been described previously (e.g., [18, 20, 38, 39]). New data from pollen were obtained from *Almeidea* and six species of *Conchocarpus*. Pollen grains were taken from mature buds of different collections deposited at SPFR. Grains were acetolysed according to the methodology described in [40] and then mounted in glycerine jelly on glass slides. Palynological terminology followed [41] and [42].

PAUP* version 4.0b10 [43] was used for morphological analysis, with parsimony as a criterion to choose the best trees. All characters were unordered and equally weighted (Fitch parsimony, [44]). Heuristic searches were performed with tree-bisection-reconnection (TBR) branch-swapping algorithm, steepest descent option and multrees options off, 10,000 random-taxon addition replicates, and 10 trees held in each replicate. All analyses were programmed to retain only 10,000 trees. Robustness of clades was estimated using bootstrap [45] implemented in PAUP*, with 10,000 pseudoreplicates with simple taxon addition and TBR branch-swapping algorithm, with steepest descent and multrees options off. Support values for bootstrap percentage (BP) were: strong (≥ 88%), moderate (76–87%), weak (63–75%), and ambiguous (<63%).

### Molecular analysis

DNA was extracted from fresh or silica-gel dried leaves (3–5 mg) according to a modified protocol of [46]. The *rps16* intron was amplified using *rps*F> and *rps*R2< primers described in [47]. The PCR reaction volume (50 μL) contained 29μL of water, 6μL Betaine solution 5M (Sigma-Aldrich), 5μL of *Taq*Buffer (10x, Fermentas), 4μL of MgCl_2_ 25mM (Fermentas), 2.5μL of dNTP 10mM (Fermentas), 0.5μL of *Taq*Polymerase 5 U/μL (Fermentas), 1μL of each primer 10 pM (Sigma-Aldrich), and 2μL (150–200ng) of DNA sample. Thermal cycling was performed in a ESCO Swift Maxi Thermal Cycler (Hatboro, PA, USA), using initial denaturation at 99°C (10 min), followed by 33 cycles at 94°C (1min), 50°C (45s), 72°C (1min 20s), ending with an elongation at 72°C (5min).

The *trnL-trnF* region was amplified using the “c” and “f” primers described in [48]. PCR reaction volume (50 μL) contained the same proportions of the same ingredients as that used to amplify the *rps16* intron. Thermal cycling was performed using initial denaturation at 99°C (10 min), followed by 35 cycles at 94°C (30s), 56°C (30s), 72°C (45s), ending with an elongation at 72°C (3min).

Nuclear regions ITS-1 and ITS-2 were amplified using primers described in [49], more specifically the 18d> and 5.8C< for ITS-1 and 5.8D> and 28CC< for ITS-2. Thermal cycling was performed using initial denaturation at 99°C (10 min), followed by 30 cycles at 95°C (15s), 56°C (30s), 72°C (1,5min), ending with an elongation at 72°C (3min). PCR products were purified with GFX^TM^ PCR columns (Amersham Biociences, Piscataway, New Jersey, USA), following the manufacturer’s recommendations. The sequencing reaction volume was 10μL, containing 3.25μL of water, 2μL of BigDye Terminator Ready Reaction (Invitrogen), 0.5μL (10mM) of primers (same used in PCR reactions), and 4.25μL of PCR product (60–150ng of DNA). The reactions were performed for both strands (forward and reverse) in an ABI-3100 automatic sequencer (Applied Biosystems-HITACHI), using initial denaturation at 96°C (5 min), followed by 40 cycles at 96°C (30s), 50°C (30s), and 60°C (4min).

Sequences were analyzed and edited using the Biological Sequence Aligment Editor (BioEdit), v.7.1.3 [50]. Each fragment was carefully examined to verify concordance among the sites. Limits of the *trnL-trnF* region, the *rps16* intron, and ITS-1 and ITS-2 regions were determined by comparison with sequences deposited at GenBank. The automated alignments of the sequences were made with MUSCLE v.3.6 [51]. Refinements of the resulting alignment were made by eye using BioEdit software again, including trimming of initial and final blocks of the alignments in order to delete primer sequences. Indels were coded as missing data.

Parsimony analyses were made using PAUP* version 4.0b10 [43]. All characters were unordered and equally weighted (Fitch parsimony, [44]). Heuristic searches were performed with the tree-bisection-reconnection (TBR) branch-swapping algorithm with “steepest descent” and “multrees” options off, with 10,000 random-taxon addition replicates, and with 1 tree held in each replicate. Bootstrap analyses [45] were implemented to verify support for the clades using TNT 1.1 [52], with 1,000 pseudoreplicates, taxon random addition, and TBR branch-swapping algorithm. TNT software is computationally more efficient than PAUP, making a bootstrap analysis with a higher number of replicates in a shorter time. The support values for bootstrap percentage (BP) were categorized in the same way as those used in the morphological analysis: strong (≥88%), moderate (76–87%), weak (63–75%), and ambiguous (<63%).

Bayesian analyses were conducted with MrBayes version 3.01 [53], using the GTR + G as substitution model, based on the Akaike information criterion using jModelTest v.0.1.1 [54]. Searches were made in two independent runs, each with four Markov chains simultaneously, started from random trees. Ten million generations were performed, sampling a tree at every 1,000 generations and the temperature coefficient of the chain-heating scheme set to 0.20. After the exclusion of 25% of the trees corresponding to the burn-in, a majority-rule consensus tree was calculated from remaining trees. The stationary and convergence of runs, as the effective sample size (ESS) was monitored using the program Tracer v.1.6 [55]. Robustness of clades was estimated as Bayesian posterior probabilities in the same software. It was considered a well-supported clade only when PP value was higher than 0.95.

### Morphological plus molecular dataset analysis

The Incongruence Length Difference Test (ILD) [56], implemented in PAUP* with 1,000 replicates and the same parameters used for parsimony searches, was used to test first, the congruence between the two nuclear marker datasets and between the two plastid marker datasets, and then the congruence between the morphological and all molecular datasets. Analysis of the complete morphological and molecular dataset was performed using parsimony with the same parameters described for individual analyses.

### Mapping of morphological characters

The history of some morphological characters (7, 11, 13, 28, 29, 33) was traced in the majority-rule consensus tree from Bayesian analysis of the combined molecular data using Mesquite v.2.75 [57] with the ancestral reconstruction of states inferred by parsimony criterion.

## Results

### Morphological Analysis

**Fig 2:**
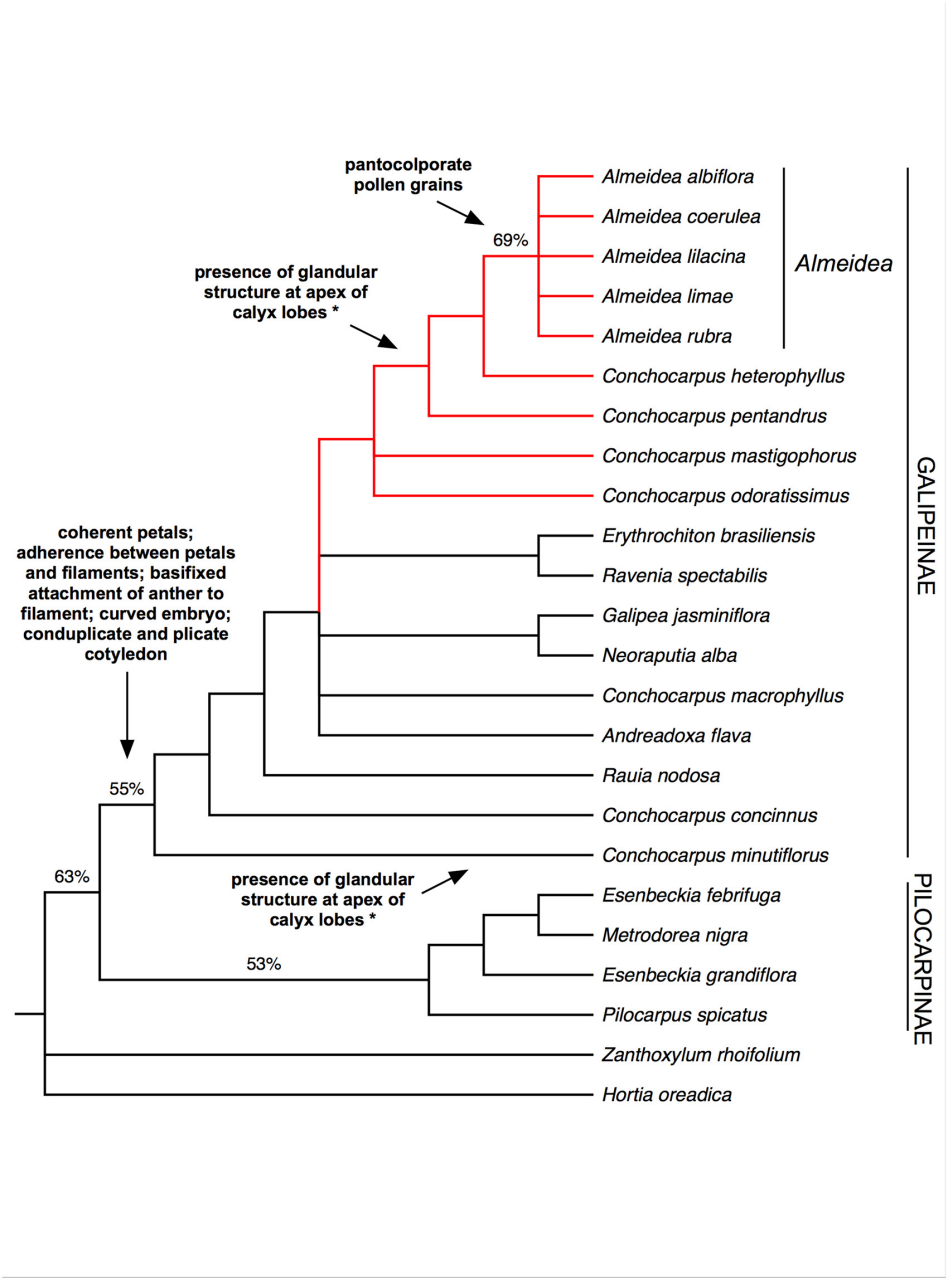
Majority-rule consensus tree of 270 equally parsimonious trees resulting from analysis of morphological data of the Galipeinae and outgroups. Support for branches is given by parsimony bootstrap percentages (≥ 50%). Possible morphological synapomorphies are indicated in the clades; * indicates possible homoplasy.

Parsimony analysis of the 35 morphological characters produced 270 equally parsimonious trees, with 128 steps (CI = 0.43, RI = 0.64, see Table 1 for a summary of all parsimony results). The strict and majority-rule (Fig 2) consensus trees resulted in similar topologies, but the clades are unsupported or weakly supported. Subtribe Galipeinae emerged as monophyletic (BP = 55%), and possible synapomorphies of the subtribe are indicated in Fig 2. *Almeidea* also appeared as monophyletic with low support (BP = 69%), with the pantocolporate (with numerous apertures) pollen grain as a synapomorphy for the genus (Fig 2). Other character state taxonomically important in *Almeidea* is the presence of glandular structure at apex of calyx lobes (present also in *Conchocarpus heterophyllus*, *C*. *pentandrus* and *C*. *minutiflorus*), indicating a possible case of homoplasy in the group (see Fig 2). However this character is discussed later with the results from molecular data, because the relationships of the morphological analysis are unsupported. *Conchocarpus* appeared as non-monophyletic, with one group of species closer than the other to *Almeidea*, to which *C*. *heterophyllus* was sister. Representatives of tribe Pilocarpinae (*Esenbeckia*, *Metrodorea*, and *Pilocarpus*) formed an unsupported clade (BP = 53%).

**Table 1:** Characteristics of the parsimony analyses.

	N° characters	N° PIC^1^	% PIC	N° trees^2^	N° steps	CI^3^	RI^4^
**Morphological analysis**	35	35	100	270	128	0.43	0.64
**Molecular analyses**							
*trnL-trnF*	1083	65	6	862	268	0.85	0.82
*rps16*	837	74	8,8	18	294	0.82	0.84
ITS-1	456	106	23,2	28	429	0.65	0.68
ITS-2	352	107	30,4	3	397	0.61	0.66
**Combined analyses**							
*trnL-trnF* + *rps16*	1920	139	7,2	1195	565	0.83	0.83
ITS-1 + ITS-2	808	213	26,3	2	834	0.62	0.41
*trnL-trnF* + *rps16* +ITS-1 + ITS-2	2728	352	12,9	12	1410	0.70	0.72
Morphology +Molecular	2763	387	14	3	1558	0.67	0.69

^1^ PIC = parsimony informative characters.

^2^ Number of equally parsimonious trees

^3^ Consistency Index

^4^ Retention Index

### Sequence characteristics

Sequences of the *trnL-trnF* region and *rps16* intron were much longer (1083 and 837 base pairs, respectively, see Table 1) than those of the nuclear regions ITS-1 and ITS-2 (456 and 352 base pairs, respectively). However, ITS-1 and ITS-2 sequences provide more polymorphic characters, with 23.2% and 30.4% of Parsimony Informative Characters (PICs), whereas *trnL-trnF* region and *rps16* intron yield 6% and 8.8% of PICs, respectively (Table 1).

### Molecular Analysis

#### Plastid markers (trnL-trnF and rps16)

**Fig 3:**
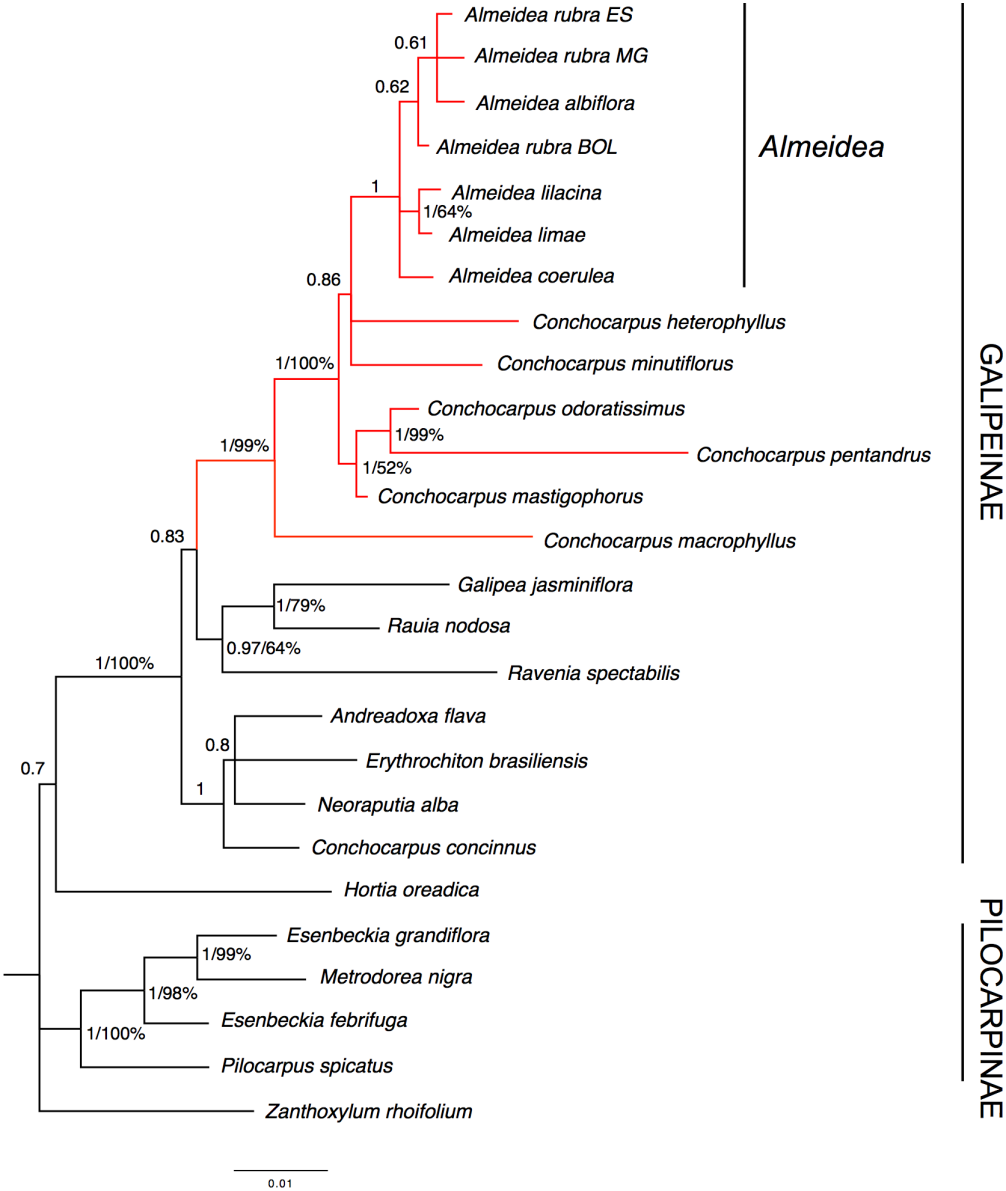
Majority-rule consensus tree estimated using Bayesian inference resulting from an analysis of the combined data from the plastid markers (*trnL-trnF* region and *rps16* intron) of Galipeinae and outgroups. Support for branches is given by Bayesian posterior probabilities and parsimony bootstrap percentages (≥ 50%). When only a number appears supporting a clade it refers to Bayesian posterior probabilities.

Given the congruence given by the result of the ILD test, the plastid markers were combined in a single matrix, as in previous studies (e.g., [13, 14]). In the majority-rule consensus trees resulting from parsimony analysis and the Bayesian analysis (Fig 3), the subtribe Galipeinae and the species of *Almeidea* appear as monophyletic groups with strong support (BP = 100%, PP = 1). The relationships among species of *Almeidea* are not well resolved, with exception of the pair, *A*. *limae* and *A*. *lilacina*, that is strongly supported in the Bayesian analysis (BP = 64%, PP = 1). *Conchocarpus* appears as non-monophyletic in both analyses (parsimony or Bayesian); six of the seven species of *Conchocarpus* in the analyses appear in a clade with *Almeidea* (BP = 99%, PP = 1), while the seventh (*C*. *concinnus*) appears with *Andreadoxa*, *Erythrochiton*, and *Neoraputia* in a strongly supported clade (PP = 1). *Galipea* and *Rauia* appear as a group (BP = 79%, PP = 1), sister to *Ravenia* (BP = 64%, PP = 0.97). Congruent with the morphological analysis, Pilocarpinae appear as a strongly supported clade (BP = 100%, PP = 1), with *Pilocarpus* sister to *Esenbeckia* and *Metrodorea*. *Hortia* appears as sister to the Galipeinae only in the Bayesian analysis, however this clade is unsupported (PP = 0.7).

#### Nuclear markers (ITS-1 and ITS-2)

**Fig 4:**
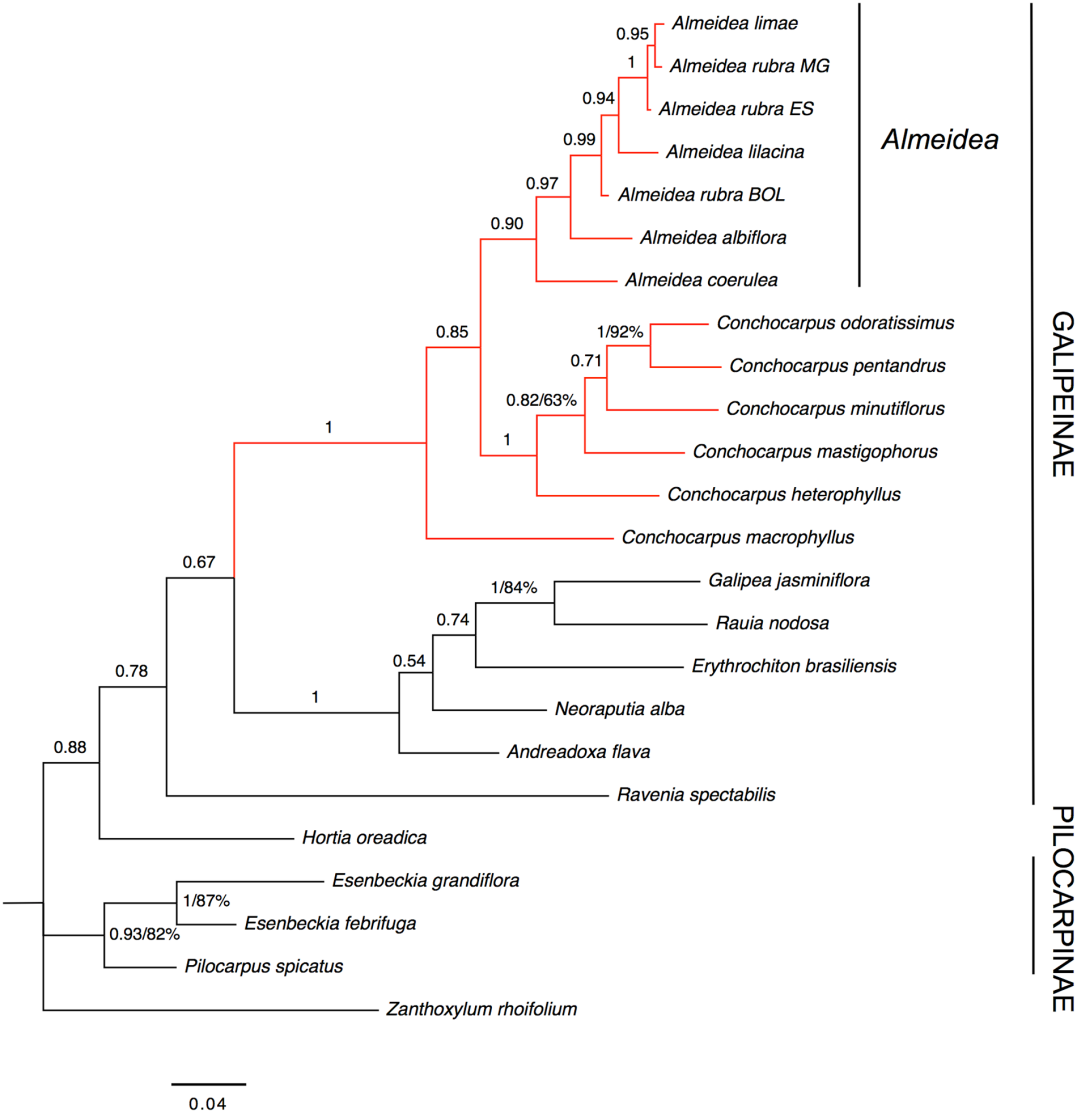
Majority-rule consensus tree estimated using Bayesian inference resulting from an analysis of the combined data from the nuclear markers (ITS-1 and ITS-2) of Galipeinae and outgroups. Support for branches is given by Bayesian posterior probabilities and parsimony bootstrap percentages (≥ 50%). When only a number appears supporting a clade it refers to Bayesian posterior probabilities.

Results of the analyses of the nuclear markers sequences are highly congruent with those obtained from the analyses of plastid markers sequences, with higher support values in most of the clades (Fig 4). The monophyly of Galipeinae and of *Almeidea* is unsupported (PP = 0.78 and PP = 0.90, respectively). On other hand, *Almeidea* and *Conchocarpus* form a clade with strong support (PP = 1), but *Conchocarpus* is non-monophyletic in relation to *Almeidea*. In the clade comprising the species of *Almeidea*, *A*. *limae* is more related to specimens of *A*. *rubra* (individuals from MG and ES, states of Brazil) than with *A*. *lilacina*, differently of the plastid markers analysis. However this relationship was supported only in the BA analysis (PP = 1), without support in the MP analysis. Congruent with the results of plastid sequences analysis, *Hortia oreadica* appears as sister to Galipeinae, and Pilocarpinae appear as monophyletic. However, *Ravenia spectabilis* appears as sister to all other Galipeinae, in an unsupported clade (PP = 0.78, without BP value), whereas in the analysis of plastid markers sequences, it appears as sister to (*Galipea*, *Rauia*), with strong support in BA analysis (BP = 64%, PP = 0.97). These different positions of *Ravenia* are supported only in Bayesian analyses of each analysis (weakly or unsupported in MP analyses), indicating a point of incongruence between nuclear and plastid datasets.

#### Combined molecular analysis

The ILD test did not find incongruences in the datasets (P > 0.05), making combining them reasonable. Besides some incongruences, as commented before, the visual inspection also revealed major congruence among the topologies resulting from the analyses of the plastid (Fig 3) and nuclear markers (Fig 4). Considering this, we decided to combine the four molecular datasets.

**Fig 5:**
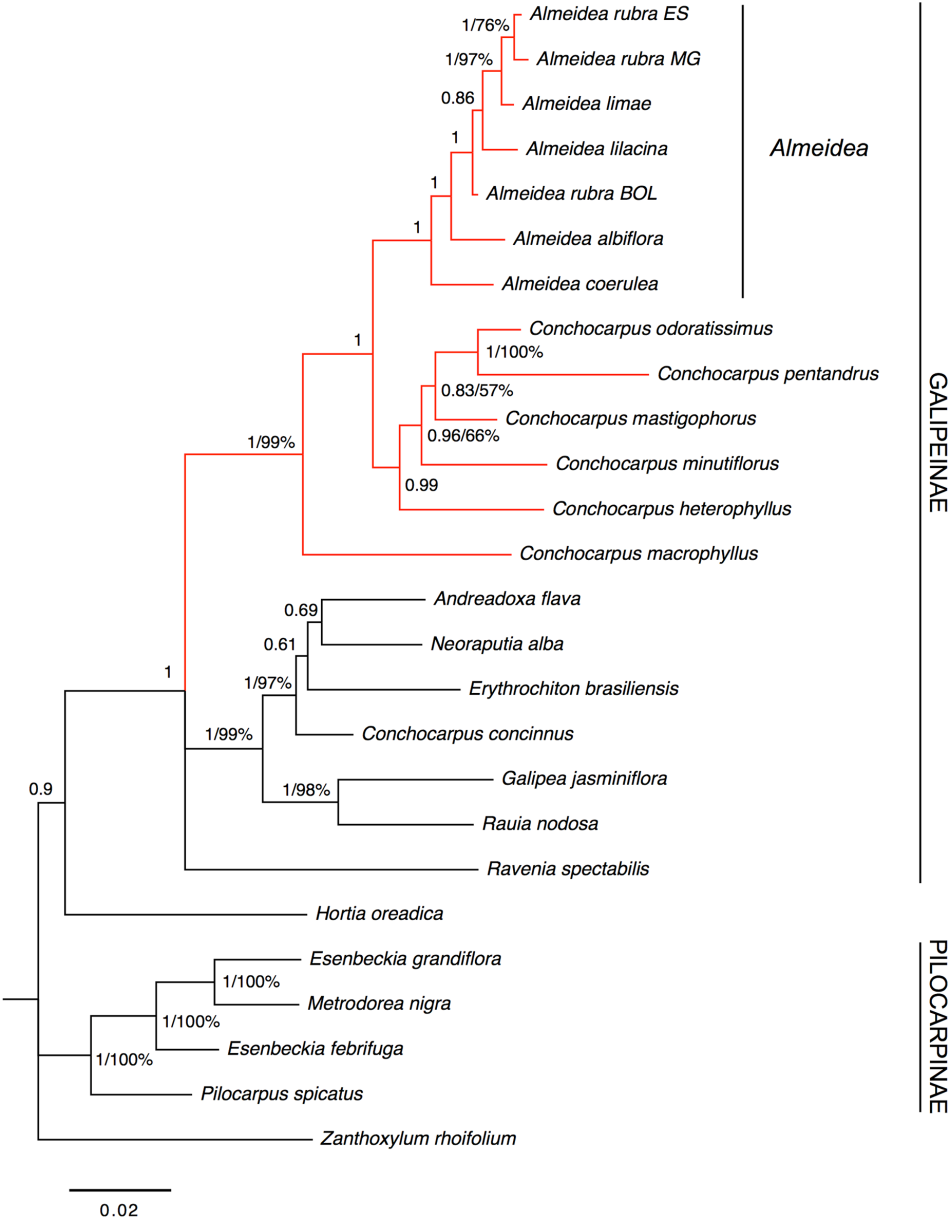
Majority-rule consensus tree estimated using Bayesian inference resulting from an analysis of the combined data from plastid and nuclear markers (*trnL-trnF*, *rps16*, ITS-1, and ITS-2) of Galipeinae and outgroups. Support for branches is given by Bayesian posterior probabilities and parsimony bootstrap percentages (≥ 50%). When only a number appears supporting a clade it refers to Bayesian posterior probabilities.

As in the analyses of plastid marker sequences and nuclear marker sequences, Galipeinae (PP = 1) and *Almeidea* (PP = 1) appear as monophyletic (Fig 5). A clade comprising the species of *Almeidea* and six of the seven species of *Conchocarpus* (*C*. *concinnus* was not sampled for ITS) has strong support (BP = 99%, PP = 1). *Ravenia* appears in an unresolved position inside Galipeinae, *Hortia oreadica* appears as sister to Galipeinae in an unsupported clade (PP = 0.9), and the subtribe Pilocarpinae is monophyletic (BP = 100%, PP = 1).

#### Combined molecular and morphological datasets analysis

The ILD test revealed that morphological and molecular datasets differed significantly (P < 0.05), indicating that these datasets should not be combined. Given, however, that some authors argue that combined datasets (whether molecular or morphological) provide the best explanatory power in analyses that involve parsimony (see [58, 59]), we decided to combine those datasets, perform a parsimony analysis, and explore the results with special caution.

**Fig 6:**
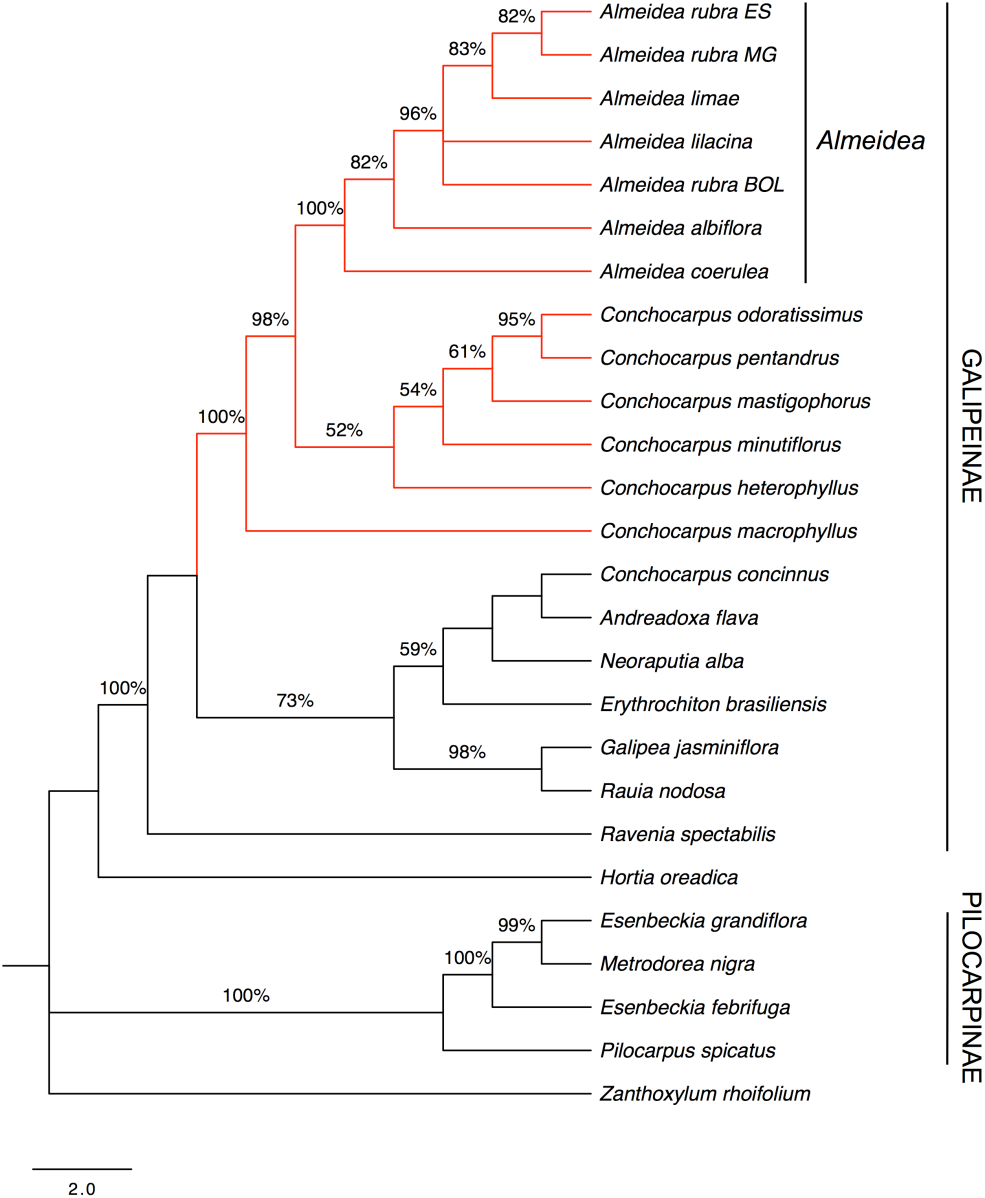
Strict consensus tree of 3 equally parsimonious trees resulting from analysis of combined morphological and molecular data of the Galipeinae and outgroups. Support for branches is given by parsimony bootstrap percentages (≥ 50%).

Topology of the strict consensus tree from the parsimony analysis (Fig 6) is largely congruent with that obtained from the combined molecular analysis (Fig 5), including the monophyly of Galipeinae (BP = 100%), Pilocarpinae (BP = 100%), and *Almeidea* (BP = 100%); also shows the close relationship of *Almeidea* with *Conchocarpus* (the latter a non-monophyletic genus) in a strongly supported clade (BP = 100%) and the position of *C*. *concinnus* in an unsupported clade with other genera of Galipeinae (BP = 59%). The pairing of *Galipea* and *Rauia* is strongly supported (BP = 98%) and they emerged as sister-group (BP = 73%) of the clade formed by *Andreadoxa*, *Erythrochiton*, and *Neoraputia* and *C*. *concinnus*. The position of *Ravenia* follows the results of the nuclear analysis, as a sister-group of the other clades in Galipeinae (BP = 100%). As in majority of other analyses, *Hortia* emerged as sister-group of Galipeinae, however without support.

## Discussion

### Utility of the molecular markers used

In the parsimony analyses, both plastid markers were more conservative than the nuclear markers and had a lower percentage of informative characters (maximum of 13% vs. 31.7%, respectively, see Table 1). On the other hand, the higher substitution rates in the nuclear markers than in the plastid markers made alignment of the sequences more subjective, not allowing the manual editing in some cases. Despite this subjectivity, found mainly for ITS-2 in this work, the trees from the analysis of the nuclear markers are better resolved at the tips of the branches and, thus, better at resolving relationships among species when compared to the trees from the plastid markers. The combined analysis of the four molecular regions includes a larger dataset and shows resolution at all levels of the trees. The strict consensus tree resulted from parsimony analysis of the combined molecular and morphological data (Fig 6) resulted in a topology highly congruent to that from the combined analysis of the four molecular regions (Fig 5). Considering the amount of data included and the resolution of the branches, we chose reconstructing the history of some important morphological characters in the majority-rule consensus tree using Bayesian inference from the combined molecular data (Fig 5) and discuss the results with basis on this tree. However, we are aware that Bayesian methods sometimes inflate support values and sometimes produce spurious clades (see [60, 61]).

### Subtribe Galipeinae

**Fig 7:**
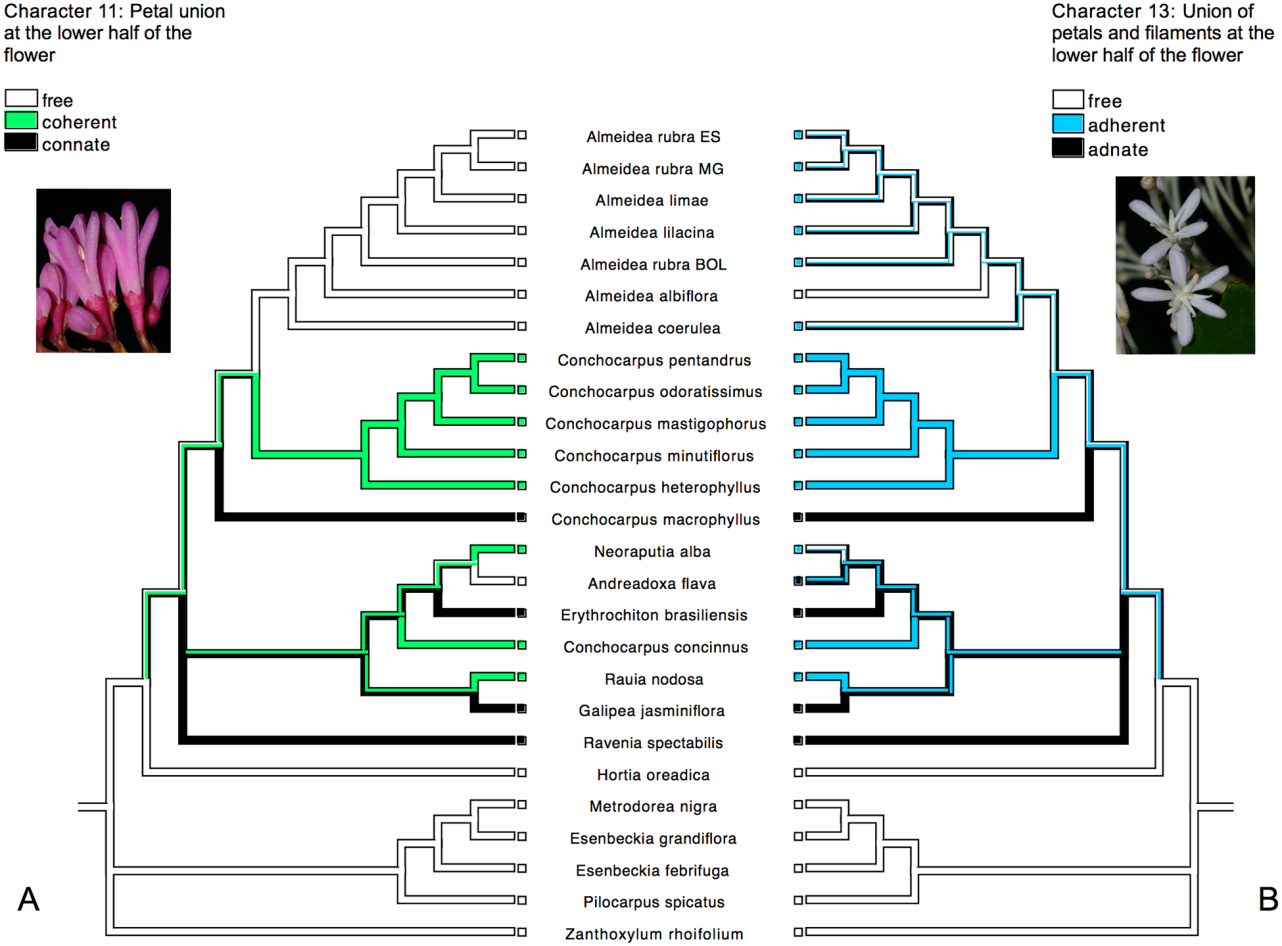
History of the morphological characters 11 and 13 traced onto the majority-rule consensus tree from Bayesian analysis of the combined molecular data: A. character 11, with photo of flower of *Almeidea rubra* (by M. Groppo), showing free petals; B. character 13, with photo of the flower of *Almeidea albiflora* (by M. Groppo), showing filaments free from the petals.

**Fig 8:**
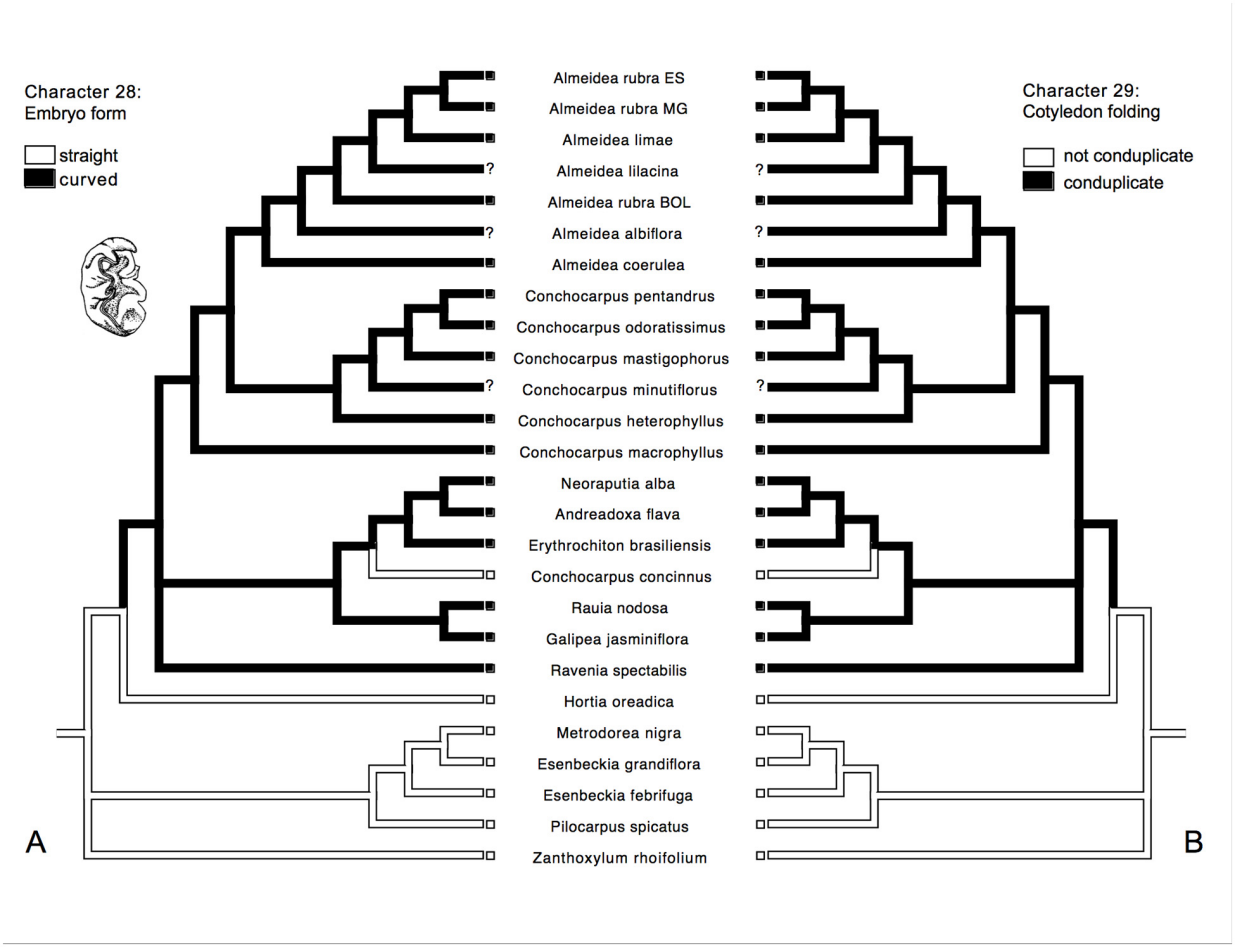
History of the morphological characters 28 and 29 traced onto the majority-rule consensus tree from Bayesian analysis of the combined molecular data: A. character 28 and B. character 29; illustration taken from [21], showing curved embryo, with conduplicate and plicate cotyledons.

All analyses showed the monophyly of Galipeinae, and some morphological characters support this clade here: the presence of coherent petals (Fig 7A), an adherence between staminal filaments and petals (Fig 7B), a basifixed attachment of anther to filament, a curved embryo (Fig 8A), conduplicate cotyledons (Fig 8B), and plicate cotyledons. Some of these characteristics was pointed previously as possible synapomorphies for the subtribe [13, 16], but were not tested before in phylogenetic analyses with focus in Galipeinae. These states are variable within Galipeinae, with some reversions occurring in some clades or taxa (e.g. free petals in *Almeidea* and *Andreadoxa*, Fig 7A), and also with some states occurring outside of the subtribe, as the curved embryo, that is also present in *Decatropis*, *Megastigma*, *Choisya* and other genera (see [5]). The variation of character states in Galipeinae also difficults the identification of morphological synapomorphies for internal clades. As an example, curved embryo with conduplicate and plicate cotyledons can be absent in some groups, as in some *Conchocarpus* (for example *C*. *concinnus*) with straight embryo and not conduplicate and not plicate cotyledons (Fig 8A and 8B). In this study, however, it is clear that appendages at the base of the anthers is synapomorphic for the clade formed by the species of *Andreadoxa*, *Erythrochiton*, *Galipea*, *Neoraputia*, *Rauia*, and *Conchocarpus concinnus*, but appendages are lacking in the two latter (perhaps through a reversion). Additionally, the clade formed by *Galipea* and *Rauia* is characterized by fruits that are almost fully syncarpic (also present in *Neoraputia*) rather than apocarpic as in all other Galipeinae.

Galipeinae exhibit a great variation in the degree to which petals and staminal filaments are united. Petals can be free without adherence to filaments (as in *Almeidea* and *Spiranthera*, not sampled here), coherent to each other and adherent to filaments (as in some *Conchocarpus*), or congenitally fused to each other and to the filaments (as in *Erythrochiton*). Fig 7A and 7B show the distribution of these characteristics in the taxa analyzed in the present study. A previous study [62] of six species of three genera of Galipeinae revealed three different patterns of fusion within and between petals and filaments: a floral tube formed by postgenital coherence and adherence of petals and filaments by interwining trichomes (as in *Conchocarpus heterophyllus* and *C*. *minutiflorus*), a tube formed by congenital fusion of petals and filaments (*Erythrochiton brasiliensis*), and a tube formed distally with the first pattern and proximally with the second pattern (*Galipea jasminiflora* and *C*. *macrophyllus*). That *C*. *macrophyllus*, *Erythrochiton* and *Galipea* appear in different clades (Fig 7A and 7B) suggests that their floral tubes, although formed by congenital fusion in all cases, are from different origins. In *Ravenia spectabilis* (not analyzed by [62]), that appears in an unresolved position with the other genera of Galipeinae, the pattern of development and fusion of petals and filaments may be of a different origin than that encountered in *C*. *macrophyllus*, *Erythrochiton* or *Galipea* or homologous to one of these, depending of its position in the subtribe, that is unresolved so far. Because this study included only nine of the 28 genera of the Galipeinae, analyses of a broader sample of the taxa are required to understand floral evolution and synapomorphic characteristics within the Galipeinae as a whole.

### *Almeidea* and *Conchocarpus*

The phylogenetic proximity between *Almeidea* and *Conchocarpus* was supported in this work by both morphological and molecular data. As said in the introduction, *Almeidea* shares many characters with several species of *Conchocarpus*, differing from them only by its free, rather than coherent or connate, petals. Although petal color was treated before as a distinguishing character, it is not an absolute; petals in *Almeidea* are usually pink or lilac but rarely white, and those of *Conchocarpus* are usually white but rarely pink or yellow.

**Fig 9:**
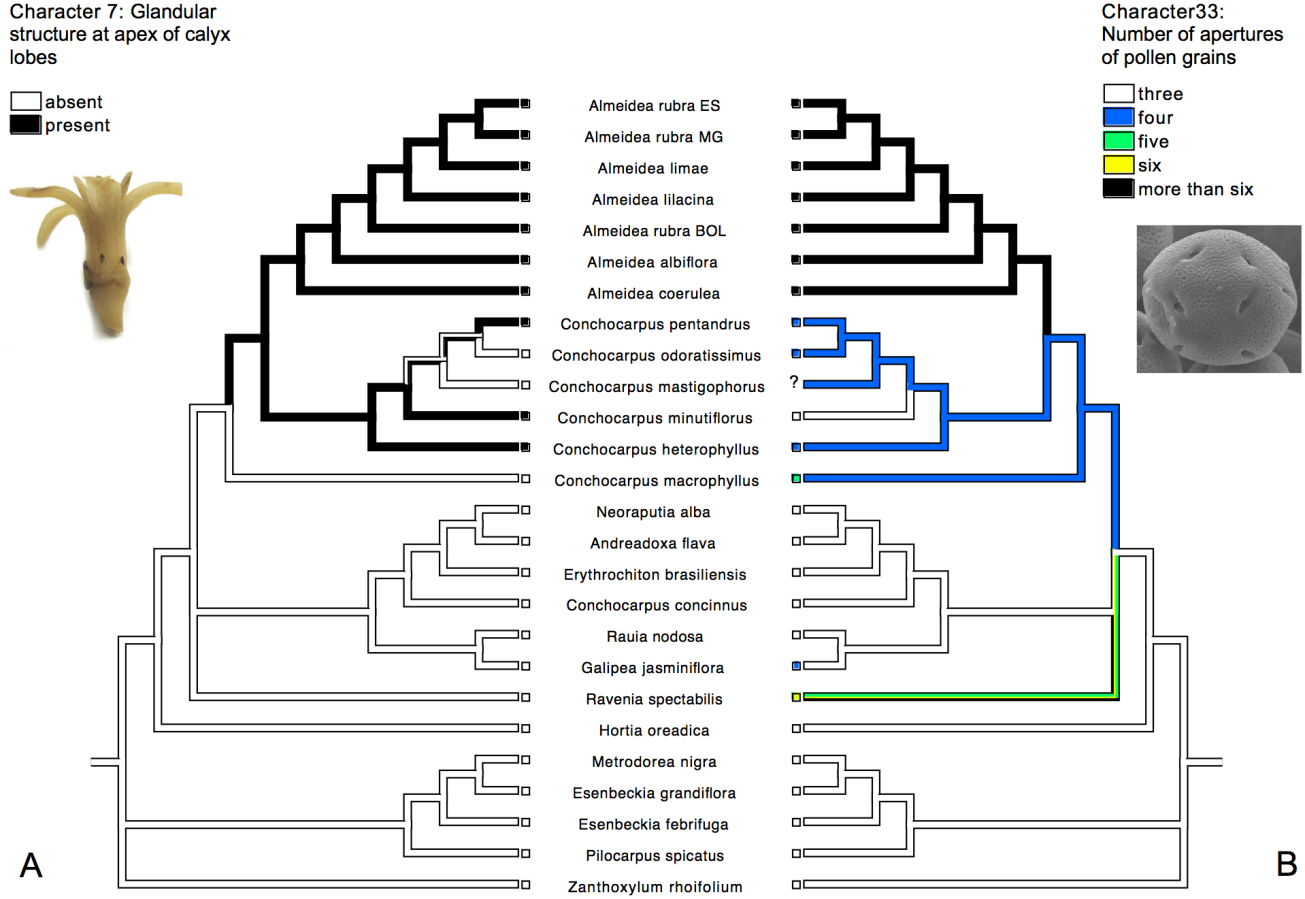
History of the morphological characters 7 and 33 traced onto the majority-rule consensus tree from Bayesian analysis of the combined molecular data: A. character 7, with photo of rehydrated flower of *Almeidea rubra* (by Juliana El Ottra), showing the presence of glandular structure in the apex of calyx lobes; B. character 33, with photo of the pantocolporate pollen grain of *Almeidea albiflora* (taken from [20]).

Due to their morphological similarity, species of *Almeidea* have been traditionally recognized as a natural genus with no doubts about its circumscription (see [9, 11, 21]). Results of this molecular study have corroborated this view by showing that the group is monophyletic. Its monophyly is further supported by the presence of pantocolporate pollen grains (Fig 9B), a unique feature among the Neotropical Rutaceae. Another feature common to all species of *Almeidea* is the presence of a glandular structure, interpreted as a nectary [21], in the apex of each calyx lobes (Fig 9A); this feature was observed also in *Conchocarpus heterophyllus*, *C*. *minutiflorus*, and *C*. *pentandrus*. This character possibly represents a synapomorphy of the clade comprising *Almeidea* and its sister species of *Conchocarpus* as showed in Fig 9A. Free petals and filaments that are not adherent to the petals are present not only in the species of *Almeidea*, but also in many genera of Rutaceae outside of the Galipeinae, and are here considered to be reversions in *Almeidea* (Fig 7A and 7B). However it is important to mention that a slightly adherence between petals and filaments was observed in some individuals of species of *Almeidea* (with exception of *A*. *albiflora*), what can only be adequately tested with anatomical studies.

The relationship between the species of *Almeidea* is unresolved in morphological analysis and some incongruences occurred between the molecular datasets analyses. However it become clear the phylogenetic proximity between *A*. *limae*, *A*. *lilacina* and *A*. *rubra*, this last one non-monophyletic because of the specimen from Bolivia, that emerged separated from Brazilian specimens. These results are in agreement with the morphological similarities between these three species, observed especially in vegetative and fruiting materials. These similarities will be detailed in another work (Bruniera et al., unpublished), with the formal recognition of a single species for *A*. *limae*, *A*. *lilacina* and *A*. *rubra*.

Contrasted with the nomenclatural stability in *Almeidea*, the species now recognized as *Conchocarpus* (see [16]) have a history of taxonomic and nomenclatural changes, as can be seen in [16] (p. 257–258), who considered *Conchocarpus* polymorphic. The position of *C*. *concinnus* in a clade separate from that of the other species of the genus corroborates the view of *Conchocarpus* as a non-monophyletic group. *C*. *concinnus* shares with four other species of *Conchocarpus* (not sampled here), which are also endemic to the Brazilian Atlantic Rain Forest, the combination of anthers broadly attached to the filaments, almost free sepals still overlapping at anthesis, a glabrous ovary, and plano-convex (not plicate) cotyledons [16]. These may best be recognized as a separate genus.

### Nomenclatural implications

Given that the species of *Almeidea* are nested in a clade of six species of *Conchocarpus* including the type species, *C*. *macrophyllus*, we propose to transfer *Almeidea* to *Conchocarpus*. The new nomenclatural combinations are presented at the end of the text. The relationships of *C*. *concinnus* and its relatives are being investigated in a major study of Galipeinae by the authors (unpublished), and a revision of the former species of *Almeidea* (now part of *Conchocarpus*) is also underway.

### Taxonomic changes

New combinations in *Conchocarpus*. Accepted names are in **bold-faced type**, synonyms in *italics*.

***Conchocarpus*** J.C. Mikan, Del. Fl. Faun. Bras. t.2. (1820). Type: *C*. *macrophyllus* J.C. Mikan
*Almeidea* A. St.-Hil., Bull. Sci. Soc. Philom. Paris 10, ser. 3: 129. 1823 [Sep]. Type: *Almeidea rubra* A. St.-Hil. (designated here).
***Conchocarpus albiflorus*** (Bruniera & Groppo) Bruniera & Groppo, comb. nov.
  = *Almeidea albiflora* Bruniera & Groppo, Brittonia 63: 282. 2011.
***Conchocarpus coeruleus*** (Nees & Mart.) Bruniera & Groppo, comb. nov.
  = *Almeidea coerulea* (Nees & Mart.) A. St.-Hil., Mém. Mus. Hist. Nat 10: 394. 1824. [Mar-Apr]
  = *Almeidea caerulea* A. St.-Hil. ex G.Don, Gen. Hist. 1: 798. 1831.
  = *Aruba coerulea* Nees & Mart., Nova Acta Phys.-Med. Acad. Caes. Leop.-Carol. Nat. Cur.40: 174. 1823.
***Conchocarpus lilacinus*** (A. St.-Hil.) Bruniera & Groppo, comb. nov.
  = *Almeidea lilacina* A. St.-Hil., 10, ser. 3: 130. 1823 [Sep].
***Conchocarpus limae*** (I.M.Silva) Bruniera & Groppo, comb. nov.
  = *Almeidea limae* I.M.Silva, Bradea 4(46): 362.1987.
***Conchocarpus rubrus*** (A. St.-Hil.) Bruniera & Groppo, comb. nov.
  = *Almeidea rubra* A. St.-Hil. Bull. Sci. Soc. Philom. Paris, 10, ser. 3: 130. 1823. [Sep]

## Supporting Information

S1 Table Matrix of morphological data. (DOCX)

S1 Text Voucher information and GenBank accession numbers for sequences produced in this study and those previously published. Herbarium acronyms follow [24]. Genbank accession numbers are in the following order: *trnL-trnF*, *rps16*, ITS-1, ITS-2. The superscript * refers to [13] where the sequence was first published.
(DOCX)

S2 Text Vouchers examined for the morphological analysis. Herbaria acronyms follow [24].
(DOCX)

S3 Text List of characters and character states used in the morphological analysis. (DOCX)
