# Supplementary material for: Almeidea A. St.-Hil. Belongs to Conchocarpus J.C. Mikan (Galipeinae, Rutaceae): Evidence from Morphological and Molecular Data, with a First Analysis of Subtribe Galipeinae
Source: PLoS One. 2015 May 7;10(5):e0125650. doi: 10.1371/journal.pone.0125650 (PMC4423776; doi:10.1371/journal.pone.0125650)
Supplement: S1 Table — (DOCX) [file pone.0125650.s001.docx]

**Matrix of morphological data:** ? = missing data; x = non aplicable

|  |  |  |  |  |  |  |  |  |  |  |  |  |  |  |  |  |  |  |  |  |  |  |  |  |  |  |  |  |  |  |  |  |  |  |  |
| --- | --- | --- | --- | --- | --- | --- | --- | --- | --- | --- | --- | --- | --- | --- | --- | --- | --- | --- | --- | --- | --- | --- | --- | --- | --- | --- | --- | --- | --- | --- | --- | --- | --- | --- | --- |
| **Taxa/Characters** | **1** | **2** | **3** | **4** | **5** | **6** | **7** | **8** | **9** | **10** | **11** | **12** | **13** | **14** | **15** | **16** | **17** | **18** | **19** | **20** | **21** | **22** | **23** | **24** | **25** | **26** | **27** | **28** | **29** | **30** | **31** | **32** | **33** | **34** | **35** |
|  |  |  |  |  |  |  |  |  |  |  |  |  |  |  |  |  |  |  |  |  |  |  |  |  |  |  |  |  |  |  |  |  |  |  |  |
| ***A. albiflora*** | 0 | 2 | 0 | 0 | ? | 0 | 1 | 1 | x | 0 | 0 | 0 | 0 | 2 | 2 | 0 | 2 | 0 | 0 | 0 | 0 | 0 | 0 | 0 | 0 | 1 | 2 | ? | ? | ? | ? | ? | 4 | 0 | 0 |
| ***A. coerulea*** | 0 | 2 | 0 | 0 | 2 | 0 | 1 | 1 | x | 0 | 0 | 0 | 0;1 | 0;2;3 | 0;1;2 | 0 | 2 | 0 | 0 | 0 | 0 | 0 | 0 | 0 | 0 | 0;1 | 0 | 1 | 1 | 1 | 1 | 1 | 4 | 0 | 0 |
| ***A. lilacina*** | 0 | 2 | 0 | 0 | 2 | 0 | 1 | 1 | x | 0 | 0 | 0 | 0;1 | 0;3 | 0;1 | 0 | 2 | 0 | 0 | 0 | 0 | 0 | 0 | 0 | 0 | 1 | 0 | ? | ? | ? | ? | ? | 4 | 0 | 0 |
| ***A. limae*** | 0 | 2 | 0 | 0;1 | 2 | 0 | 1 | 0 | 0 | 0 | 0 | 0 | 0;1 | 0;3 | 0;1;2 | 0 | 2 | 0 | 0 | 0 | 0 | 0 | 0 | 0 | 0 | 1 | 0 | 1 | 1 | 1 | 1 | 1 | 4 | 0 | 0 |
| ***A. rubra*** | 0 | 2 | 0 | 0;1 | 2 | 0 | 1 | 1 | x | 0 | 0 | 0 | 0;1 | 0;1;2;3 | 0;1;2;3 | 0 | 2 | 0 | 0 | 0 | 0 | 0 | 0 | 0 | 0 | 1 | 0 | 1 | 1 | 1 | 1 | 1 | 4 | 0 | 0 |
| ***C. concinnus*** | 0 | 2 | 0 | 0 | 0 | 1 | 0 | 0 | 1 | 0 | 1 | 0 | 1 | 1 | 3 | 1 | 0 | 1 | 0 | 0 | 2 | 1 | 0 | 0 | 0 | 0 | 0 | 0 | 0 | 0 | 0 | 1 | 0 | 3 | 0 |
| ***C. heterophyllus*** | 0 | 1 | 0 | 0 | 0 | 0 | 1 | 1 | x | 0 | 1 | 0 | 1 | 2 | 2 | 1 | 2 | 0 | 0 | 0 | 0 | 1 | 0 | 0 | 0 | 0 | 0 | 1 | 1 | 1 | 1 | 1 | 1 | 0 | ? |
| ***C. macrophyllus*** | 0 | 2 | 0 | 0 | 0 | 0 | 0 | 1 | x | 0 | 2 | 0;1 | 2 | 1 | 3 | 1 | 1 | 1 | 0 | 0 | 2 | 1 | 0 | 0 | 0 | 0 | 0 | 1 | 1 | 1 | 1 | 1 | 1;2 | 0 | 0 |
| ***C. mastigophorus*** | 0 | 1 | 0 | 1 | 0 | 0 | 0 | 0 | 0 | 0 | 1 | 0 | 1 | 1 | 3 | 1 | 1 | 0 | 0 | 0 | 0 | 0 | 0 | 0 | 0 | 0 | 0 | 1 | 1 | 1 | 1 | 1 | ? | ? | ? |
| ***C. minutiflorus*** | 0 | 2 | 0 | 1 | 0 | 0 | 1 | 0 | 0 | 0 | 1 | 0 | 1 | 1;2 | 2;3 | 1 | 0 | 0 | 0 | 0 | 0 | 1 | 0 | 0 | ? | ? | ? | ? | ? | ? | ? | ? | 0 | 4;5 | 0 |
| ***C. odoratissimus*** | 0 | 2 | 0 | 1 | 0 | 0 | 0 | 0 | 0 | 0 | 1 | 1 | 1 | 1;2 | 2;3 | 1 | 1 | 0 | 0 | 0 | 1 | 0 | 0 | 0 | 0 | 0 | 0 | 1 | 1 | 1 | 1 | 1 | 1 | 0;3 | 0 |
| ***C. pentandrus*** | 0 | 2 | 0 | 1 | 0 | 0 | 1 | 1 | x | 0 | 1 | 0 | 1 | 0;2;3 | 0;1;2 | 1 | 1 | 0 | 0 | 0 | 0 | 0 | 0 | 0 | 0 | 0 | 0 | 1 | 1 | 1 | 1 | 1 | 1 | 0;1 | 0 |
| ***A. flava*** | 0 | 2 | 0 | 0 | ? | 0 | 0 | 0 | 0 | 0 | 0 | 0 | 1;2 | 1 | 5 | 1 | 2 | 1 | 1 | 0 | 0 | 1 | 0 | 0 | 0 | 1 | 2 | 1 | 1 | 1 | 1 | 1 | 0 | 0 | 4 |
| ***E. brasiliensis*** | 0 | 2 | 2 | 1 | 1 | 0 | 0 | 1 | x | 0 | 2 | 1 | 2 | 0;3 | 0;1 | 1 | 2 | 0 | 1 | 0 | 1 | 1 | 0 | 0 | 1 | 0 | 0 | 1 | 1 | 1 | 1 | 0 | 0 | 0 | 5 |
| ***G. jasminiflora*** | 0 | 1 | 0 | 0 | ? | 0 | 0 | 0 | 1 | 0 | 2 | 0;1 | 2 | 1 | 5;6 | 1 | 2 | 0 | 1 | 0 | 0 | 1 | 1 | 1 | 1 | 0 | 0 | 1 | 1 | 1 | 1 | 1 | 0;1 | 0 | 0 |
| ***N. alba*** | 0 | 2 | 2 | 0 | ? | 0 | 0 | 0 | 0 | 0 | 1 | 0 | 0;1 | 1 | 3 | 1 | 1 | 0 | 1 | 0 | 0 | 1 | 1 | 1 | 1 | 0 | 0 | 1 | 1 | 1 | 1 | 1 | 0 | 0 | 0 |
| ***R. nodosa*** | 0 | 1 | 0 | 0;1 | ? | 0 | 0 | 0 | 0 | 0 | 1 | 0 | 1 | 1 | 3;4 | 1 | 2 | 1 | 0 | 0 | 1 | 0 | 1 | 1 | 0 | 0 | 0 | 1 | 1 | 0 | 0 | 1 | 0 | 4 | 3 |
| ***R. spectabilis*** | 1 | 1 | 2 | 1 | 2 | 1 | 0 | 0 | 0 | 0 | 2 | 1 | 2 | 1 | 3 | 1 | 0 | 0 | 0 | 0 | 1 | 1 | 0 | 0 | 0 | 0 | 0 | 1 | 1 | 0 | 1 | 1 | 2;3 | 0 | 0 |
| ***E. febrifuga*** | 1 | 1 | 1 | 0 | 0 | 1 | 0 | 0 | 0 | 0 | 0 | 0 | 0 | 0 | 0 | 0 | 0 | 0 | 0 | 0 | 1 | 1 | 1 | 1 | 0 | 0 | 1 | 0 | 0 | 0 | 0 | 1 | 0 | 4 | 0 |
| ***E. grandiflora*** | 0 | 2 | 1 | 0 | 0 | 1 | 0 | 0 | 0 | 0 | 0 | 0 | 0 | 0 | 0 | 0 | 0 | 1 | 0 | 0 | 1 | 1 | 1 | 1 | 0 | 0 | 1 | 0 | 0 | 0 | 0 | 1 | 0 | 4 | 0 |
| ***M. nigra*** | 1 | 1 | 0 | 0 | 1 | 0 | 0 | 1 | x | 1 | 0 | 0 | 0 | 0 | 0 | 0 | 0 | 0 | 0 | 0 | 1 | 0 | 1 | 1 | 0 | 0 | 1 | 0 | 0 | 0 | 0 | 1 | 0 | ? | 2 |
| ***P. spicatus*** | 0;1 | 3 | 1 | 1 | 1 | 1 | 0 | 0 | 0 | 1 | 0 | 0 | 0 | 0 | 0 | 0 | 0 | 0 | 0 | 0 | 0 | 1 | 0 | 0 | 0 | 0 | 1 | 0 | 0 | 0 | 0 | 1 | 0 | 4 | 0 |
| ***H. oreadica*** | 0 | 3 | 0 | 0 | 0 | 0 | 0 | 0 | 0 | 1 | 0 | 0 | 0 | 0 | 0 | 0 | 2 | 0 | 0 | 1 | 0 | 0 | 1 | 1 | 1 | 0 | 0 | 0 | 0 | 0 | 1 | 0 | 0 | 4 | 3 |
| ***Z. rhoifolium*** | 0 | 0 | 0 | 0 | ? | 0 | 0 | 0 | 0 | 0 | 0 | 0 | 0 | 0 | 0 | 0 | 0 | 0 | 0 | 1 | 0 | 0 | 0 | 0 | 1 | 1 | 1 | 0 | 0 | 0 | ? | 0 | 0 | 3;4 | 1 |
|  |  |  |  |  |  |  |  |  |  |  |  |  |  |  |  |  |  |  |  |  |  |  |  |  |  |  |  |  |  |  |  |  |  |  |  |
