# Supplementary material for: Almeidea A. St.-Hil. Belongs to Conchocarpus J.C. Mikan (Galipeinae, Rutaceae): Evidence from Morphological and Molecular Data, with a First Analysis of Subtribe Galipeinae
Source: PLoS One. 2015 May 7;10(5):e0125650. doi: 10.1371/journal.pone.0125650 (PMC4423776; doi:10.1371/journal.pone.0125650)
Supplement: S1 Text — Herbarium acronyms follow [24]. Genbank accession numbers are in the following order: trnL-trnF, rps16, ITS-1, ITS-2. The superscript * refers to [13] where the sequence was first published. (DOCX) [file pone.0125650.s002.docx]

**Voucher information and GenBank accession numbers for sequences produced in this study and those previously published.** Herbarium acronyms follow [24]. Genbank accession numbers are in the following order: *trnL-trnF*, *rps16*, ITS-1, ITS-2. The superscript * refers to [13] where the sequence was first published.

***Almeidea albiflora*** Bruniera & Groppo, Brazil, Espírito Santo, Cachoeiro de Itapemirim, *Groppo 1852* (SPFR), KP866579, KP866600, KP866620, --; *Groppo 1853* (SPFR), --, --, --, KP866643. ***Almeidea coerulea*** (Nees & Mart.) A. St.-Hil ex G. Don., Brazil, Bahia, Itacaré, *Bruniera 92* (SPFR), KP866580, KP866601, KP866621, KP866644. ***Almeidea lilacina*** A. St.-Hil., Brazil, São Paulo, Campinas, *Bruniera 122* (SPFR), KP866581, KP866602, KP866622, KP866645. ***Almeidea limae*** I.M.Silva, Brazil, Rio de Janeiro, Guapimirim, *Bruniera 79* (SPFR), KP866582, KP866603, KP866623, KP866646. ***Almeidea rubra* ES** A. St.-Hil., Brazil, Espírito Santo, Santa Teresa, *Bruniera 105* (SPFR), --, KP866604, KP866624, KP866647; *Bruniera 108* (SPFR), KP866584, --, --, --. ***Almeidea rubra* MG** A. St.-Hil., Brazil, Minas Gerais, Carangola, *Bruniera 109* (SPFR), KP866585, --, --, --; *Bruniera 110* (SPFR), --, KP866605, KP866626, KP866648. ***Almeidea rubra* BOL** A. St.-Hil.**,** Bolivia, La Paz, Franz Tamayo, *Cayola 2094* (SPF), KP866583, --, KP866625, --. ***Andreadoxa flava*** Kallunki, Brazil, Bahia, Ilhéus, *Pirani 4973* (SPF), --, KP866606, KP866627, --; Brazil, Bahia, Ilhéus*, Groppo 1562* (SPFR), KP866586, --, --, KP866649. ***Conchocarpus concinnus*** Kallunki, Brazil, Bahia, Ilhéus, *Groppo 1610* (SPFR), KP866587, KP866607, --, --. ***Conchocarpus heterophyllus*** (A. St.-Hil.) Kallunki & Pirani, Brazil, Espírito Santo, Linhares, *Groppo 999* (SPF), KP866588, KP866611, KP866628, KP866653. ***Conchocarpus macrophyllus*** J.C.Mikan, Brazil, Espírito Santo, Santa Teresa, *Groppo 985* (SPF), KP866589, KP866612, --, --. Brazil, Bahia, Jussari, *Groppo 1571* (SPFR), --, --, KP866629, --. ***Conchocarpus mastigophorus*** Kallunki, Brazil, Bahia, Uruçuca, *Groppo 1589* (SPFR), KP866590, KP866608, KP866630, KP866650. ***Conchocarpus minutiflorus*** Groppo & Pirani, Brazil, Espírito Santo*,* Santa Teresa, *Groppo 1617* (SPFR), KP866591, KP866609, KP866631, KP866654. ***Conchocarpus odoratissimus*** (Lindl.) Kallunki & Pirani, Brazil, Rio de Janeiro, Tijuca*, Groppo 1540* (SPFR), KP866592, KP866610, KP866632, KP866651. ***Conchocarpus pentandrus*** (A. St.-Hil.) Kallunki & Pirani, Brazil, Minas Gerais, Santana do Riacho, *Pirani 4996* (SPF), EU853789^*^, EU853735^*^, --, --; Brazil, São Paulo, Ribeirão Preto*, Groppo 1262* (SPF), --, --, KP866633, KP866652. ***Erythrochiton brasiliensis*** Nees & Mart., Brazil, Espírito Santo, Santa Teresa*,* *Groppo 975* (SPF), --, KP866613, --, --. Brazil, Bahia, Jussari, *Groppo 1570* (SPFR), KP866593, --, --, KP866655. ***Esenbeckia febrifuga*** (A. St.-Hil.) Juss. ex. Mart., Brazil, Bahia, Santa Cruz da Vitória, *Groppo 1577* (SPFR), KP866594, KP866614, KP866634, KP866657. ***Esenbeckia grandiflora*** Mart., Brazil, São Paulo, São Paulo, *Groppo 1149* (SPF), EU853795^*^, KP866615, --, --. Brazil, Espírito Santo, Rio Bananal, *Groppo 1827* (SPFR), --, --, KP866635, KP866656.

***Galipea jasminiflora*** (A. St.-Hil.) Engl., Brazil, São Paulo, Ribeirão Preto, Bruniera 118 (SPFR), KP866595, KP866616, KP866636, KP866658. ***Hortia oreadica*** Groppo, Kallunki & Pirani, Brazil, Goiás, Rio Verde, *Groppo 458* (SPF), EU853803^*^, EU853750^*^, KP866637, KP866659. ***Metrodorea nigra*** A. St.-Hil., Brazil, São Paulo, São Paulo, *Groppo 1111* (SPF), EU853809^*^, EU853757^*^, --, --. ***Neoraputia alba*** (Nees & Mart.) Emmerich ex Kallunki, Espírito Santo, Santa Teresa*,* *Groppo 979* (SPF), --, KP866617, --, --; Brazil, Espírito Santo, Santa Teresa*, Groppo 1624* (SPFR), KP866596, --, KP866638, KP866660. ***Pilocarpus spicatus*** A. St.-Hil., Brazil, Minas Gerais, Santana do Riacho, *Pirani 4995* (SPF), --, EU853761^*^, --, --; Brazil, Rio de Janeiro, Jardim Botânico*, Groppo 1520* (SPFR), KP866597, --, KP866639, KP866661. ***Rauia nodosa*** (Engl.) Kallunki, Brazil, Bahia, Una, *Pirani 4975* (SPF), KP866598, KP866619, --, --; Brazil, Bahia, Jussari*, Groppo 1565* (SPFR), --, --, KP866640, KP866662. ***Ravenia spectabilis*** (Lindl.) Engl., Brazil, Rio de Janeiro, Jardim Botânico, Groppo 1514 (SPFR), KP866599, KP866618, KP866641, --. ***Zanthoxylum rhoifolium*** Lam., Brazil, São Paulo, São Paulo, *Groppo 1145* (SPF), EU853773^*^, EU853720^*^, KP866642, KP866663.
