# Supplementary material for: Almeidea A. St.-Hil. Belongs to Conchocarpus J.C. Mikan (Galipeinae, Rutaceae): Evidence from Morphological and Molecular Data, with a First Analysis of Subtribe Galipeinae
Source: PLoS One. 2015 May 7;10(5):e0125650. doi: 10.1371/journal.pone.0125650 (PMC4423776; doi:10.1371/journal.pone.0125650)
Supplement: S2 Text — Herbaria acronyms follow [24]. (DOCX) [file pone.0125650.s003.docx]

**Vouchers examined for the morphological analysis.** Herbarium acronyms follow [24].

***Almeidea albiflora*** Bruniera & Groppo: D. Araujo 6012 (GUA, NY), M. Groppo 1851^*,1^, 1852, 1853, 1861 (SPFR), J. Kallunki 595 (NY, SPF), J.R. Pirani 1130 (SPF, SPFR); ***Almeidea coerulea*** (Nees & Mart.) A. St.-Hil.: A.M. Amorim 927, 1299, 1669, 2649^*^ (CEPEC), C.P. Bruniera 88, 92, 93, 97, 98 (SPFR), A.M. de Carvalho 3422, 4128, 4558 (CEPEC), R.M. Harley 18427^*^ (CEPEC, NY), J.G. Jardim 1770 (CEPEC, NY), 4100 (CEPEC), J.A. Kallunki 494 (NY, SPF), 682 (NY), S. Mori 11749 (CEPEC, NY), T.S. Nunes 995 (HUEFS, SPF), J.L. Paixão 231 (CEPEC), J.R. Pirani 2747 (SPF), S.C. de Sant’Ana 599 (CEPEC), W. Thomas 7457, 8084, 9200, 9757 (NY), 9525, 9796, 11856 (CEPEC, NY), 9775 (NY, SPF); ***Almeidea lilacina*** A. St.-Hil.: E. de Amaral s.n. ^*^ (SP), J.E.A. Bertoni 309 (SPF), C.P. Bruniera 121, 122, 124 (SPF), R.B. Cardamone 176 (SPF), J. Kallunki 596 (NY, SPF), L.S. Leoni 1471^*^ (GFJP, SPF), K. Santos 5^*^ (SPF), Serviço Florestal do Estado de São Paulo 35^*^ (SP), D. Sucre II.222 (RB), J. Vasconcellos Neto 6055 (RB); ***Almeidea limae*** I.M.Silva: J.M.A. Braga 4783^*^ (RB), C.P. Bruniera 79^*^, 80^*^, 81 (SPFR), L.C. Giordano 107 (RB), R. Guedes 2232, 2233 (RB, SPF), H.C. de Lima 2215^*^ (RB, SPFR), 4380 (RB, SPF), G. Martinelli 9904^*^, 9999 (RB), 10119 (RB, SPF, SPFR), M. Peron 897 (RB); ***Almeidea rubra*** A. St.-Hil.: A. Araujo-M. 1987 (MO, NY), 2032, 2168, 2191 (MO, SPF), A.C. Brade 18435 (NY, RB, SPFR), C.P. Bruniera 142, 151 (SPFR), L. Cayola 1769, 2094 (MO, SPF), L.V. Costa s.n.^*^ (BHCB, SPF), V. Demuner 942 (MBML, SPFR), G.L. Farias 118 (NY), D. Fernandes 205^*^ (RB), P. Fiaschi 2275 (CEPEC), G.S. França 485 (BHCB), G. Hatschbach 42571 (MBM, SPF), 33752 (MBM, RB, SP, UPCB), 61481 (MBM, RB), 68883 (ALCB, MBM, UPCB), N. Helme 127 (LPB, NY), J.G. Jardim 2090 (CEPEC, NY), H.C. de Lima 5289 (RB), 6090 (NY, RB), M. Macia 7629 (MO, NY), R. Marquete 2856 (RB), J. Ordones s.n. (BHZB, SPFR), S.V.A. Pessoa 1113 (RB, SPFR), J.R. Pirani 3395 (RB, SPF), 3415 (SPF), L.P. de Queiroz 10706^*^ (HUEFS, SPF), R. Seidel 2241 (LPB, NY), D. Sucre 10098 (RB), L.G. Temponi 10 (BHCB), W.W. Thomas 10248 (NY), P.C. Vinha 1205 (SPF); ***Andreadoxa flava*** Kallunki: M. Groppo 1562 (SPFR), T.S. dos Santos 4587, 4588 (CEPEC); ***Conchocarpus concinnus*** Kallunki: C.P. Bruniera 102 (SPFR), M. Groppo 1610, 1613 (SPFR), J.G. Jardim 254 (CEPEC), J.A. Kallunki 686 (CEPEC, NY), L.A. Mattos Silva 1480, 1690 (CEPEC, SPF), J.R. Pirani 2943 (SPF); ***Conchocarpus heterophyllus*** (A. St.-Hil.) Kallunki & Pirani: D. Cardoso 769 (HUEFS), M.A.A. Costa 97, 98 (ALCB), G. Hatschbach 71549 (CEPEC, MBM), J.A. Kallunki 394^*^ (CEPEC, NY), M.A.B.L. Machado 122 (ALCB, MAC), E. Melo 3217 (HUEFS), J.R. Pirani 2893 (HUEFS, SPF), L.P. Queiroz 9908 (HUEFS), T.S. Santos 1849^*^, 2334^*^ (NY); N.P. Taylor 1577 (CEPEC); ***Conchocarpus macrophyllus*** J.C.Mikan: R.A.X. Borges 836^*^ (CEPEC), C.P. Bruniera 83, 85^*^ (SPFR), D. Cardoso 786 (CEPEC, HUEFS), P. Fiaschi 1283 (CEPEC), M. Groppo 1515, 1532, 1571, 1574^*^, 1627 (SPFR), W.W. Thomas 12358, 13842^*^, 14498 (CEPEC, NY); ***Conchocarpus mastigophorus*** Kallunki: A.M. de Carvalho 3421 (CEPEC, SPF), 3590 (ALCB, CEPEC, SPF), 4401 (CEPEC, SPF), M. Groppo 1589, 1590 (SPFR), J.A. Kallunki 495 (NY, SPF), 527 (NY, SPF, SPFR)^2^, J.R. Pirani 2346 (NY, SPF), W.W. Thomas 9035 (NY, SPF), 9744 (NY, SPF), 10400 (NY, SPF) ^2^; ***Conchocarpus minutiflorus*** Groppo & Pirani: V. Demuner 543 (MBML, SPF), M. Groppo 1617^*^, 1618^*^ (SPFR), L. Kollmann 4457 (MBML, SPF), J.R. Pirani 4931 (SPF, SPFR); ***Conchocarpus odoratissimus*** (Lindl.) Kallunki & Pirani: A.P. Duarte 5495 (RB, SPF), M. Groppo 1540, 1541^*^, 1542 (SPFR), J.A. Kallunki 714 (NY, SPF), H.C. de Lima 4259, 4278 (RB, SPF), J.R. Pirani 3417, 3440, 3566 (SPF); ***Conchocarpus pentandrus*** (A. St.-Hil.) Kallunki & Pirani: L.C. Bernacci 11 (SPFR), M. Groppo 1262^*^, 1449^*^ (SPFR), O. Kotchetkoff-Henriques 649^*^ (SPFR); ***Erythrochiton brasiliensis*** Nees & Mart.: M. Groppo 1569, 1570, 1626 (SPFR), J.G. Jardim 1474, 1873 (CEPEC, SPFR), R.S. Pinheiro 2340 (CEPEC), T.S.S. 1639 (CEPEC); ***Esenbeckia febrifuga*** (A. St.-Hil.) Juss. ex. Mart.: F.A. Cloclet s.n. (FUEL, SPFR), M. Groppo 1577 (SPFR), A.B. Gusman 1531 (SPFR), G. Hatschbach 48544 (MBM, SPF), O. Kotchetkoff-Henriques 645 (SPFR), L.P. de Queiroz 2177 (HUEFS), E.R. Salviani 88 (HPL, SPFR), J.M. Silva 2528 (MBM, SPF), R.A.G. Viani s.n. (FUEL, SPFR), A.E. Yoshida s.n. (FUEL, SPFR), P.C. Zampa s.n. (CESJ, SPF); ***Esenbeckia grandiflora*** Mart.: A.M. de Carvalho 727 (CEPEC), M.C. Ferreira 822 (HRB, SPF), A.M. Giulietti 1664 (CEPEC, HUEFS), M. Groppo 1522, 1578, 1759, 1827 (SPFR), M.L. Guedes 194, 7392, 8062, 8131 (ALCB), J.G. Jardim 1982 (CEPEC), A. Leal-Costa 845 (ALCB), J. Paula-Souza 5260 (ESA, SPFR), R.S. Pinheiro 2143 (CEPEC), J.R. PIrani 2001 (SPF), R.R. Rodrigues s.n. (ESA, SPFR); ***Galipea jasminiflora*** (A. St.-Hil.) Engl.: A.B. Gusman 205 (SPFR), O. Kotchetkoff-Henriques 609, 610, 611, 612 (SPFR), W. Mantovani s.n. (ESA, SPFR); ***Hortia oreadica*** Groppo, Kallunki & Pirani: P.G. Delprete 9425 (SPF), F.T. Farah 380, 449, 569 (ESA, SPF), G.F. Faria 09 (CEN, SPF), M. Groppo 458 (SPF), 846 (SPF), 1355 (SPFR), B.A.S. Pereira 3396 (SPF), G. Pereira-Silva 7927 (CEN, SPF), M.R. Silva 1020 (SPF); ***Metrodorea nigra*** A. St.-Hil.: G.F. Arbocz 1995 (HPL, SPFR), C.P. Bruniera 115 (SPFR), J.S. Carneiro 556 (FUEL, SPF), A.L. Costa s.n. (ALCB), M. Groppo 1111 (SPF), 1529 (SPFR), A.B. Gusman 1485 (SPFR), O. Kotchetkoff-Henriques 651, 652, 653 (SPFR), R.S.C.R. Lima 27 (SPF, SPSF), Pinheiro 1251 (CEPEC), C.V. Roderjan 198 (MBM, SPF), T.S. dos Santos 763, 1206 (CEPEC), E.S. da Silva 30 (CEPEC), W.W. Thomas 10851 (CEPEC); ***Neoraputia alba*** (Nees & Mart.) Emmerich: L.J. Alves s.n. (ALCB), P.I.S. Braga s.n. (BHCB, SPF), M. Gomes 26 (RB, SPF), M. Groppo 1624, 1625 (SPFR), J. Kallunki 611 (NY, SPF), L. Kollmann 5686 (MBML, SPF), M.A. Lopes 102 (SPF), J.R. Pirani 3524 (SPF), C.E. Ramos 236 (ALCB, SPF), V. de Souza 52, 264 (SPF), D. Sucre 8898 (RB, SPF); ***Pilocarpus spicatus*** A. St.-Hil.: P.D. Carvalho 299 (HUEFS), J. Costa 3535 (HUEFS), J.M. Gonçalves 72 (HUEFS), M. Groppo 1520, 1521 (SPFR), R.M. Harley 55009 (HUEFS), A. Miranda 250 (ALCB), L. Paganucci 138 (ALCB), L. Passos 5719 (ALCB), L.P. de Queiroz 5255 (HUEFS), N. Roque 1797 (ALCB), E. Saar 5251 (ALCB), V.C. Souza 29905 (ESA, HUEFS); ***Rauia nodosa*** (Engl.) Kallunki: R.M. Harley 22055 (SPF), J.G. Jardim 191 (CEPEC, HUEFS), J.A. Kallunki 370, 748 (NY, SPF), L.A. Mattos-Silva 4570 (HUEFS, UESC), O.J. Pereira 2228, 2477 (SPF), J.R. Pirani 2754, 3075, 3493, 4734 (SPF), L.V. Rosa 257 (SPF), W.W. Thomas 9198 (NY, SPF); ***Ravenia spectabilis*** (Lindl.) Engl.: J. Bertoldo 01 (RB, SPF), M. Groppo 1514 (SPFR); ***Zanthoxylum rhoifolium*** Lam.: R. Barros 3260 (SPFR, TEPB), C.P. Bruniera 158 (SPFR), A.M de Carvalho s.n. PCD 2180 (ALCB, HUEFS), W. Ganev 2251, 2570 (HUEFS), R.M. Harley 54804, 54837 (HUEFS), G. Hatschbach 47891 (HUEFS, MBM), M.M.M. Lopes 812 (CEPEC, HUEFS), E.B. Miranda 95 (HUEFS), J. Paula-Souza 4464, 4881 (ESA, SPFR), L.P. de Queiroz 5550, s.n. PCD 4025 (ALCB, HUEFS), C.E. Ramos 324 (ALCB).

* specimen utilized for pollen morphology

^1^ holotype

^2^ isotype

PCD: Projeto Chapada Diamantina
