# Supplementary material for: Almeidea A. St.-Hil. Belongs to Conchocarpus J.C. Mikan (Galipeinae, Rutaceae): Evidence from Morphological and Molecular Data, with a First Analysis of Subtribe Galipeinae
Source: PLoS One. 2015 May 7;10(5):e0125650. doi: 10.1371/journal.pone.0125650 (PMC4423776; doi:10.1371/journal.pone.0125650)
Supplement: S3 Text — (DOCX) [file pone.0125650.s004.docx]

**List of characters and character states used in the morphological analysis.**

1. Leaf arrangement

0. alternate

1. opposite

2. Leaf division

0. pinnately compound

1. palmately compound

2. 1-foliate

3. simple

3. Type of inflorescence ramification

0. Thyrse

1. Indeterminate

2. Determinate

4. Secondary rachis of inflorescence

0. present (> 5.0 mm)

1. absent (≤ 5.0 mm)

5. Calyx aestivation

0. quincuncial

1. valvate

2. imbricate

6. Calyx lobe margins at anthesis

0. not overlapping

1. overlapping

7. Glandular structure at apex of calyx lobes

0. absent

1. present

8. Calyx duration

0. persistent in fruit

1. deciduous

9. Calyx expansion in fruit

0. not expanded

1. expanded (at least twice length)

10. Corolla aestivation

0. imbricate

1. valvate

11. Petal union at the lower half of the flower

0. free

1. coherent (joined through interwining trichomes)

2. connate

12. Union of filaments

0. free

1. connate

13. Union of petals and filaments at the lower half of the flower (after anthesis)

0. free

1. adherent ( joined by intertwining trichomes of the margins of the petals and filaments)

2. adnate

14. Number of fertile stamens

0. Five

1. Two

2. Three

3. Four

15. Number of staminodia

0. zero

1. one

2. two

3. three

4. four

5. five

6. six

16. Attachment of anther to filament

0. dorsifixed

1. basifixed

17. Anther outline

0. ovoid (proportion 3:2)

1. narrowly-ovoid (proportion 2:1)

2. linear-oblong (proportion 3:1)

18. Anther apex

0. not modified

1. sterile

19. Gynophore

0. absent

1. present

20. Height of disc relative to ovary

0. shorter

1. taller

21. Trichomes on ovary (after anthesis)

0. absent

1. present

22. Attachment of style to ovary

0. style arising at apex of carpel (ovary attenuate)

1. style arising on axial edge of carpel below its apex (ovary umbilicate)

23. Axial union of carpels in fruit before dehiscence

0. absent

1. present

24. Lateral union of carpels in fruit before dehiscence

0. absent

1. present

25. Texture of seed testa

0. not crustaceous (tears when dissected)

1. crustaceous (cracks when dissected)

26. Surface of seed testa

0. not smooth

1. smooth

27. Hilum shape

0. circular

1. linear

2. oblate

28. Embryo form

0. straight

1. curved

29. Cotyledon folding

0. not conduplicate

1. conduplicate

30. Cotyledon plication

0. absent

1. present

31. Cotyledon in cross-section

0. plano-convex

1. flat

32. Endosperm of mature seed

0. present

1. absent or inconspicuous

33. Number of apertures of pollen grains

0. three

1. four

2. five

3. six

4. numerous (more than six)

34. Pollen shape

0. spheroidal (including oblate-spheroidal and prolate-spheroidal; P/E = 0.88< P/E < 1.14)

1. oblate (0.5< P/E <0.75)

2. suboblate (0.75< P/E <0.88)

3. subprolate (1.14< P/E <1.33)

4. prolate (1.33< P/E <2.0)

5. perprolate (P/E > 2.0)

* P/E = ratio of the lengh of the polar (P) and equatorial (E) axis (P/E)

35. Pollen exine

0. reticulate

1. reticulate-striate

2. striate

3. perforate

4. gemmate

5. baculate
